# The Research Topic Defines “Noise” in Social Media Data – a Response from the Authors

**DOI:** 10.2196/jmir.6824

**Published:** 2017-06-02

**Authors:** Yoonsang Kim, Jidong Huang, Sherry Emery

**Affiliations:** ^1^ NORC at the University of Chicago Health Media Collaboratory Chicago, IL United States; ^2^ Georgia State University Health Management and Policy Atlanta Georgia

**Keywords:** automated tweets, noise, social media data

We provide a response to Allem and Ferrara [1], who recently commented on our article, “Garbage in Garbage Out: Data Collection, Quality Assessment and Reporting Standards for Social Media Data Use in Health Research, Infodemiology and Digital Disease Detection,” which was published in JMIR in February 2016 [2]. In their comment, published in JMIR in August 2016, entitled “The importance of Debiasing Social Media Data to Better Understand E-Cigarette-Related Attitudes and Behaviors,” Allem and Ferrara discuss the importance of removing bias in social media data. They claim that automated tweets are noise that injects bias into the data, and thus should be removed before applying the framework we proposed [1]. We believe they misunderstood our intent. In addition, their discussion misinterprets the key messages of our article; the implication of their comments, which suggests that automated tweets are garbage, is highly misleading. A formal response is provided here to articulate accurately the main focus of our article and present a different view about the “noise” in social media data.

The objective of our paper was “to develop and apply a framework of social media data collection and quality assessment, and to propose a reporting standard,” as stated in the abstract. The e-cigarette-related tweet data were used as “a real-world example” to demonstrate how to apply this framework to develop a search filter, and how to estimate the measures of data quality under different conditions. The objective of our paper was not to understand e-cigarette-related attitudes and behaviors expressed on Twitter. 

The definition of the “noise” in social media data by Allem and Ferrara, as any tweets produced from an account identified as a social bot, is narrow and oversimplifying, and may even be misleading in some cases. Organic and commercial tweets are not isolated in the Twittersphere. Many organic tweets are retweets or replies to commercial tweets, of which a large number is generated by bots. Whether automated contents generated by bots should be considered as noise depends on the research topic at hand. Although it may be important to remove bot tweets and focus solely on organic contents for certain research topics, it is equally important to measure the amount of these bot tweets and the content of (mis)information in these tweets for many other research topics [3]. For example, a study that examines the commercial advertising on e-cigarette should include the tweets generated by bots. The automated social media messages are not unique to the topic of e-cigarettes. For many other research topics, including other tobacco products, pharmaceutical products, dietary supplements, etc., automatically-generated marketing content is common. In fact, one of the studies that Allem and Ferrara cited to justify removing automated tweets discussed the value of “understanding the effect of promotionally marketing vaporization products” on social media using “cyborgs to mimic organic users” because of their importance to public health and policy [4]. This underscores the importance of being able to identify and quantify such automated messages in order to understand their impact on the marketplace and individual attitudes, beliefs and behaviors. 

Allem and Ferrara also briefly discussed the inherent bias in social media data due to the fact that social media users are not a representative sample of the general population. However, this itself does not limit the value of social media data, and it can be used as an advantage to study hard-to-reach populations such as young adults, and ethnic, racial, and sexual minorities. Social media can serve as a good alternative or complementary data source to understand behavior and intentions among these understudied and hard-to-reach groups. 

Removing automated contents and applying other approaches to remove noise can be considered in the stage of developing search filters if it is deemed appropriate for the research topics in study. However, it is not a necessary component to be considered for all research using social media data. This point underscores the main thesis of our paper: that clear disclosure about data cleaning and processing (e.g. whether bot tweets are included or not) is important.
